# A Proteomic Study on Molecular Mechanism of Poor Grain-Filling of Rice (*Oryza sativa* L.) Inferior Spikelets

**DOI:** 10.1371/journal.pone.0089140

**Published:** 2014-02-21

**Authors:** Zhixing Zhang, Hong Zhao, Jun Tang, Zhong Li, Zhou Li, Dongmei Chen, Wenxiong Lin

**Affiliations:** 1 College of Life Science, Fujian Agricultural and Forestry University, Fuzhou, Fujian, China; 2 Fujian Provincial Key Laboratory of Agroecological Processing and Safety Monitoring, Fujian Agriculture and Forestry University, Fuzhou, Fujian, China; Cankiri Karatekin University, Turkey

## Abstract

Cultivars of rice (*Oryza sativa* L.), especially of the type with large spikelets, often fail to reach the yield potential as expected due to the poor grain-filling on the later flowering inferior spikelets (in contrast to the earlier-flowering superior spikelets). The present study showed that the size and grain weight of superior spikelets (SS) was greater than those of inferior spikelets (IS), and the carbohydrate supply should not be the major problem for the poor grain-filling because there was adequate amount of sucrose in IS at the initial grain-filling stage. High resolution two-dimensional gel electrophoresis (2-DE) in combination with Coomassie-brilliant blue (CBB) and Pro-Q Diamond phosphoprotein fluorescence stain revealed that 123 proteins in abundance and 43 phosphoproteins generated from phosphorylation were significantly different between SS and IS. These proteins and phosphoproteins were involved in different cellular and metabolic processes with a prominently functional skew toward metabolism and protein synthesis/destination. Expression analyses of the proteins and phosphoproteins associated with different functional categories/subcategories indicated that the starch synthesis, central carbon metabolism, N metabolism and cell growth/division were closely related to the poor grain-filling of IS. Functional and expression pattern studies also suggested that 14-3-3 proteins played important roles in IS poor grain-filling by regulating the activity of starch synthesis enzymes. The proteome and phosphoproteome obtained from this study provided a better understanding of the molecular mechanism of the IS poor grain-filling. They were also expected to be highly useful for improving the grain filling of rice.

## Introduction

Rice (*Oryza sativa* L.) is one of the world’s most important staple crops. It is essential for global food security, especially in the populous Asian and African regions [1]. The grains of rice grow on the spikelets, which can be classified as SS or IS according to their location on a branch and the time of flowering [2], [3]. In general, SS are on the apical primary branches, while IS on the proximal secondary branches on a rice plant. By comparison, SS flower earlier and fill faster with larger and heavier grains than IS [2]–[4]. The poor grain-filling of IS on rice cultivars, especially for the “super” varieties developed recently that bear numerous spikelets per panicle, has become a subject for study, as it not only negatively affects the final yield but also the milling and quality of the rice [5]–[7].

The grain-filling of rice is largely a process of starch accumulation, since the starch contributes 90% of the dry weight of an unpolished mature grain [8]. However, it has been reported that the carbohydrates may not be the only limiting factor [3], [5], [9]. Low activities of the enzymes that convert sucrose to starch, such as sucrose synthase (SuSase), adenosine diphosphateglucose pyrophosphorylase (AGPase), starch synthase (StSase), and starch branching enzyme (SBE), might also contribute to the low filling rate and weight of the grains on IS [10]–[12]. In addition, a low abscisic acid (ABA)/ethylene ratio and cytokinins and indole acetic acid (IAA) contents were also considered important in this regard [4], [13], [14]. Exogenously applied ABA or mild water stress, which resulted in a significant increase of grain ABA content at the early grain-filling stage, significantly stimulated the grain-filling of IS [12], [15].

A complex biological process, filling of a rice grain involves 21,000 genes including 269 that are closely related to various physiological and biochemical pathways [16]. Thus, to understand the process thoroughly, not only the conventional physiological and biochemical means, but also the molecular methods, must be applied. Ishimaru *et al.* showed that the gene expressions of vacuolar invertase (*INV3*), SuSase (*RSus3*), and AGPase (*AGPL1* and *AGPS2*) were much lower in IS than SS [11]. Recently, applying the high-throughput sequencing technology, differences in gene expressions between the rice spikelets was unveiled. A DNA microarray and real-time PCR analysis showed a group of genes relating to the starch metabolism, and with their enhanced expression profiles, the higher transcript levels in SS than IS at the early (EGS) and mid-grain-filling stages (MGS) [17]. The expression profiles of the miRNAs showed that 189 of them were differentially expressed between SS and IS [18]. However, the genome DNA sequence can only show, to a certain degree, the possible existence of corresponding functions of genes. It cannot predict whether or when the genes can be expressed. On the other hand, proteomic profiling can provide valuable insights regarding molecular mechanisms of poor grain-filling of IS. Thus far, there is no expression patterns of the abovementioned proteins available to enable the differentiation between SS and IS at the grain-filling stages. We proposed that the grain-filling disparity stemmed from the differential expressions of relevant proteins in the temporal and space of the spikelets.

Using the proteomic approach, we aimed to differentiate the protein expressions and to determine their relationship with the poor IS grain-filling during the EGS, MGS and late grain-filling stage (LGS). A 2-DE gel-based proteomic methodology was applied. The proteomes of SS and IS were compared. The phosphoprotein regulations of SS and IS at different grain-filling stages were differentiated using Pro-Q Diamond phosphoprotein in gel stain.

## Results

### Physiological Characteristics of Grain-filling of SS and IS

**Figure 1:**
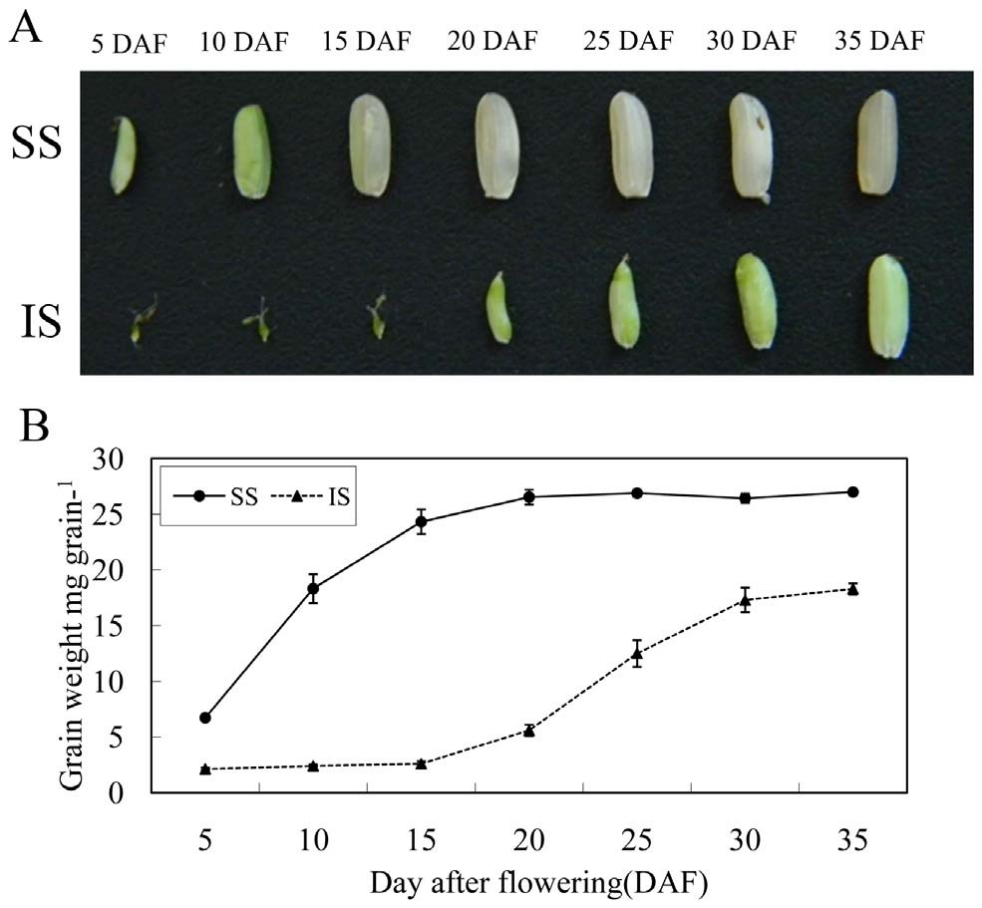
Superior spikelets (SS) and inferior spikelets (IS) in rice. A: Developmental changes in representative SS and IS B: Grain weight of SS and IS. Experiments were repeated 6 times. Error bars represent standard errors.

A developmental asynchronism was observed on the grain-fillings between SS and IS. After flowering, the size of SS increased rapidly, but the development of IS appeared stagnant from 5 to 15 DAF (Fig. 1A). The grain weight on SS increased rapidly from 5 to 20 DAF, while that on IS did not show significant increases until 20 to 35 DAF (Fig. 1B). Nonetheless, the end grain weight of IS was lower than that of SS (Fig. 1B).

**Figure 2:**
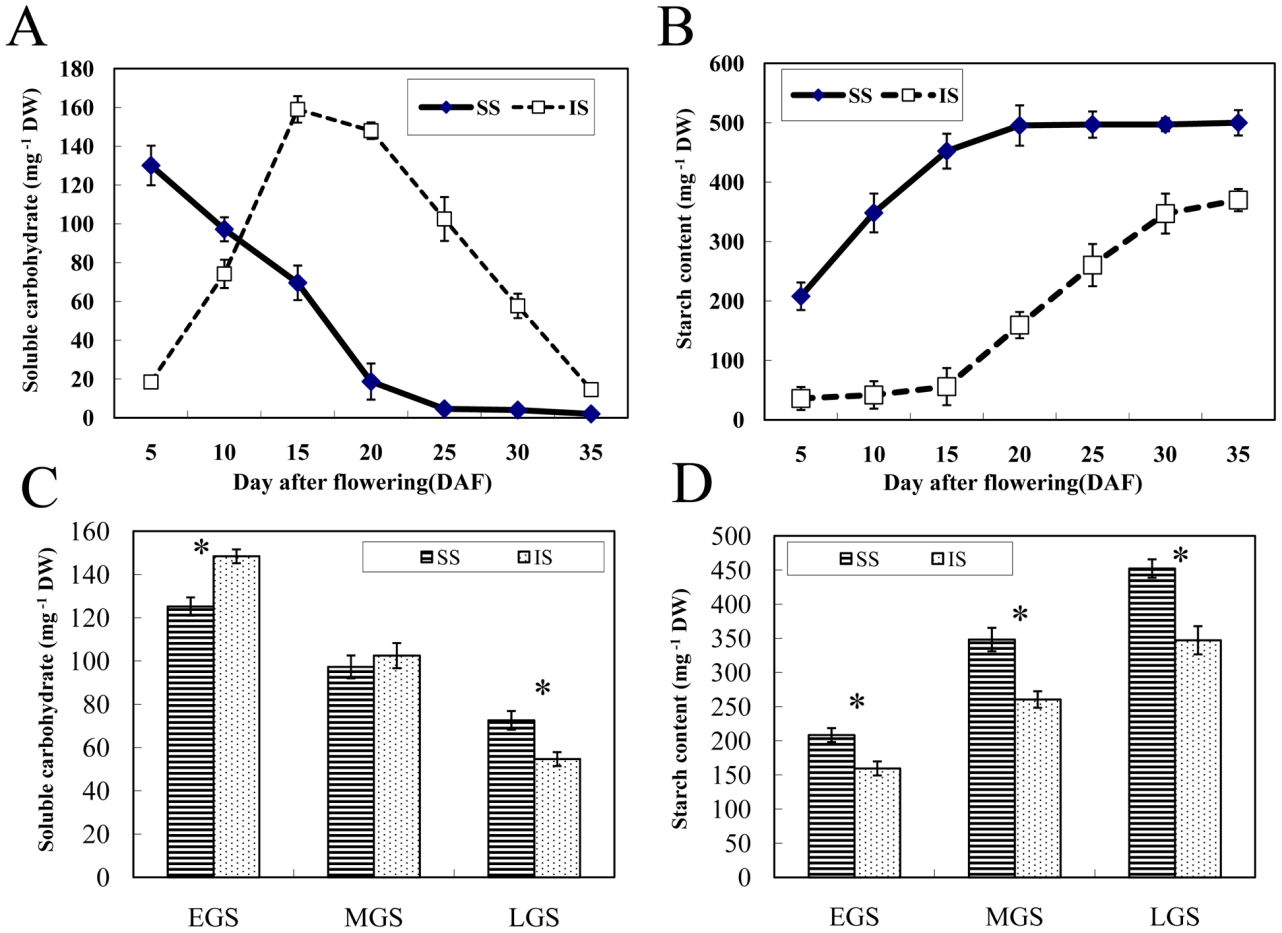
Concentrations of soluble carbohydrate and starch in grains on superior spikelets (SS) and inferior spikelets (IS) in rice of rice. A: Dynamic change of soluble carbohydrate concentration in grains on SS and IS. B: Dynamic change of starch concentration in grains on SS and IS. C: Difference of soluble carbohydrate concentration between SS and IS at three stages. D: Difference of starch concentration between SS and IS at three stages. Experiments were repeated 6 times. Error bars represent standard errors. EGS, MGS and LGS represent early, mid-, and late grain-filling stage, respectively, of endosperm development stages of SS and IS. Asterisk (*) represents significant difference (P = 0.05) between SS and IS.

The sucrose content in SS decreased after 5 DAF. In contrast, in IS, it increased rapidly after 5 DAF, peaked at 15 DAF, and decreased thereafter (Fig. 2A). Similar patterns of change on starch content and grain weight on SS and IS were observed (Fig. 1B & 2B). As shown in Fig. 2C and D, higher soluble carbohydrates contents were found on IS than SS in EGS and MGS, but lower in LGS; and, that SS had a higher starch content than IS throughout the periods.

### 2-DE Analysis and Identification of Differentially Expressed Proteins and Phosphoproteins on SS and IS

**Figure 3:**
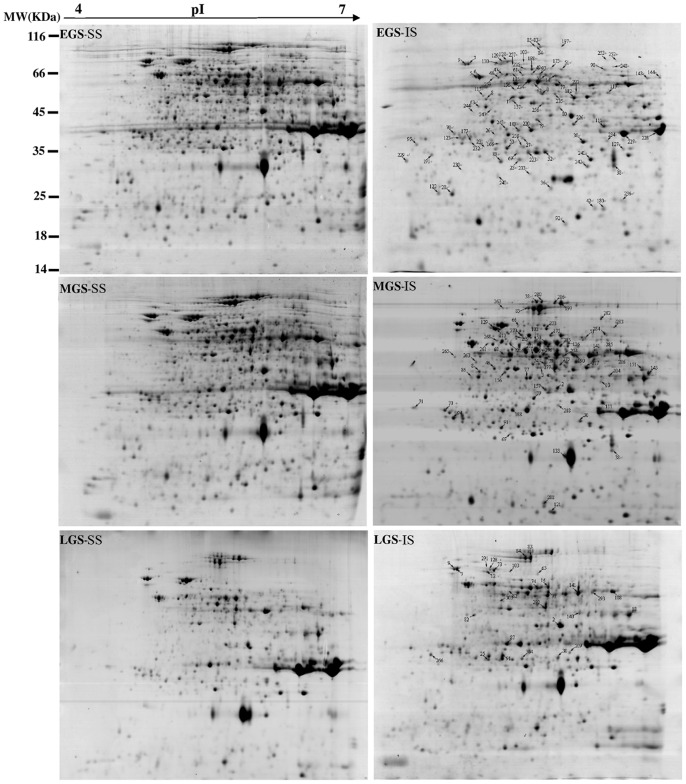
Representative 2-DE gels of proteins from superior spikelets (SS) and inferior spikelets (IS) at three grain-filling stages. Three independent replicates for superior and inferior spikelets at each stage. EGS, MGS and LGS represent early, mid-, and late grain-filling stage, respectively, of endosperm development stages of SS and IS. Proteins prepared from SS and IS at the EGS, MGS and LGS were separated by 2-DE and stained by Coomassie Brilliant Blue. The differentially expressed protein spots between SS and IS at three stages were determined according to the method described in “Materials and methods”. MW (in kilodaltons) and pI of the proteins are shown at left and top, respectively.

**Figure 4:**
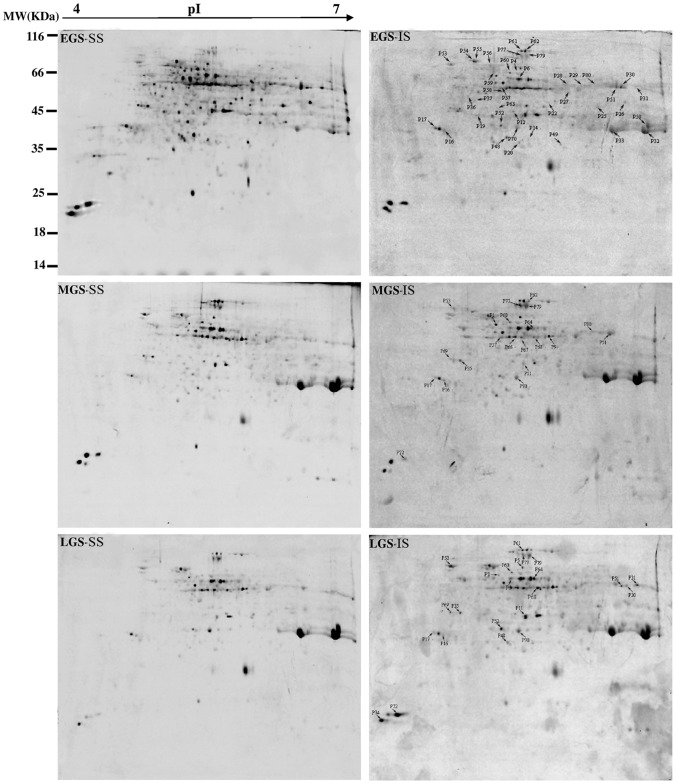
Representative 2-DE gels of phosphoproteins from superior spikelets (SS) and inferior spikelets (IS) at three grain-filling stages. Three independent replicates for SS and IS at each stage. EGS, MGS and LGS represent early, mid-, and late grain-filling stage, respectively, of endosperm development stages of SS and IS. Proteins prepared from the EGS, MGS and LGS were separated by 2-DE and stained by Pro-Q Diamond phosphoprotein. The differentially expressed phosphoprotein spots between SS and IS at three stages were determined according to the method described in “Materials and methods”. MW (in kilodaltons) and pI of the proteins are shown at left and top, respectively.

**Figure 5:**
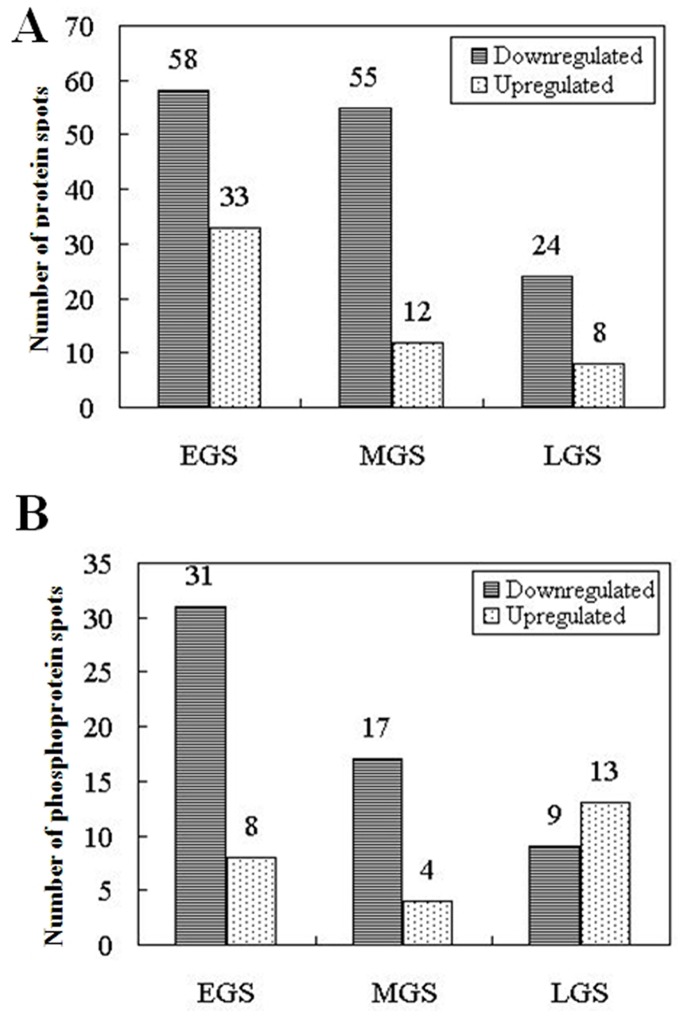
Patterns of change on differentially expressed protein spots (A) and phosphoprotein spots (B) of inferior spikelets (IS) in comparison to superior spikelets (SS). EGS, MGS and LGS represent early, mid-, and late grain-filling stage, respectively, of endosperm development stages of SS and IS.

In this study, all proteins from SS and IS were resolved and identified by using the high-resolution 2-DE followed by the CBB (Fig. 3) or Pro-Q Diamond phosphoprotein gel staining (Fig. 4). On the CBB-stained 2-DE gels (Fig. 3), 1,300±75 spots/gel in EGS, 1,350±45 spots/gel in MGS and 1,000±40 spots/gel in LGS could be visualized. It was determined that the intensities of 91 spots in EGS, 67 spots in MGS and 32 spots in LGS differed significantly (≥1.5-fold) between SS and IS. Of these protein spots, 58 (63.7%) were downregulated and 33 (36.3%) upregulated in EGS, 55 (82.1%) downregulated and 12 (17.9%) upregulated in MGS, and 24 (75%) downregulated and 8 (36.3%) upregulated in LGS for IS, as compared to SS (Fig. 5A). The 2-DE maps stained with Pro-Q Diamond resolved 455±40, 425±35 and 390±36 phosphoprotein spots/gel in EGS, MGS, and LGS, respectively (Fig. 4). On IS, a total of 39 spots in EGS, 21 spots in MGS and 22 spots in LGS were found to be differentially phosphorylated. Of these phosphoprotein spots, 31 (79.5%) were downregulated and 8 (20.5%) upregulated in EGS, 17 (81%) downregulated and 4 (19%) upregulated in MGS, and 9 (40.9%) downregulated and 8 (59.1%) upregulated in LGS (Fig. 5B).

**Figure 6:**
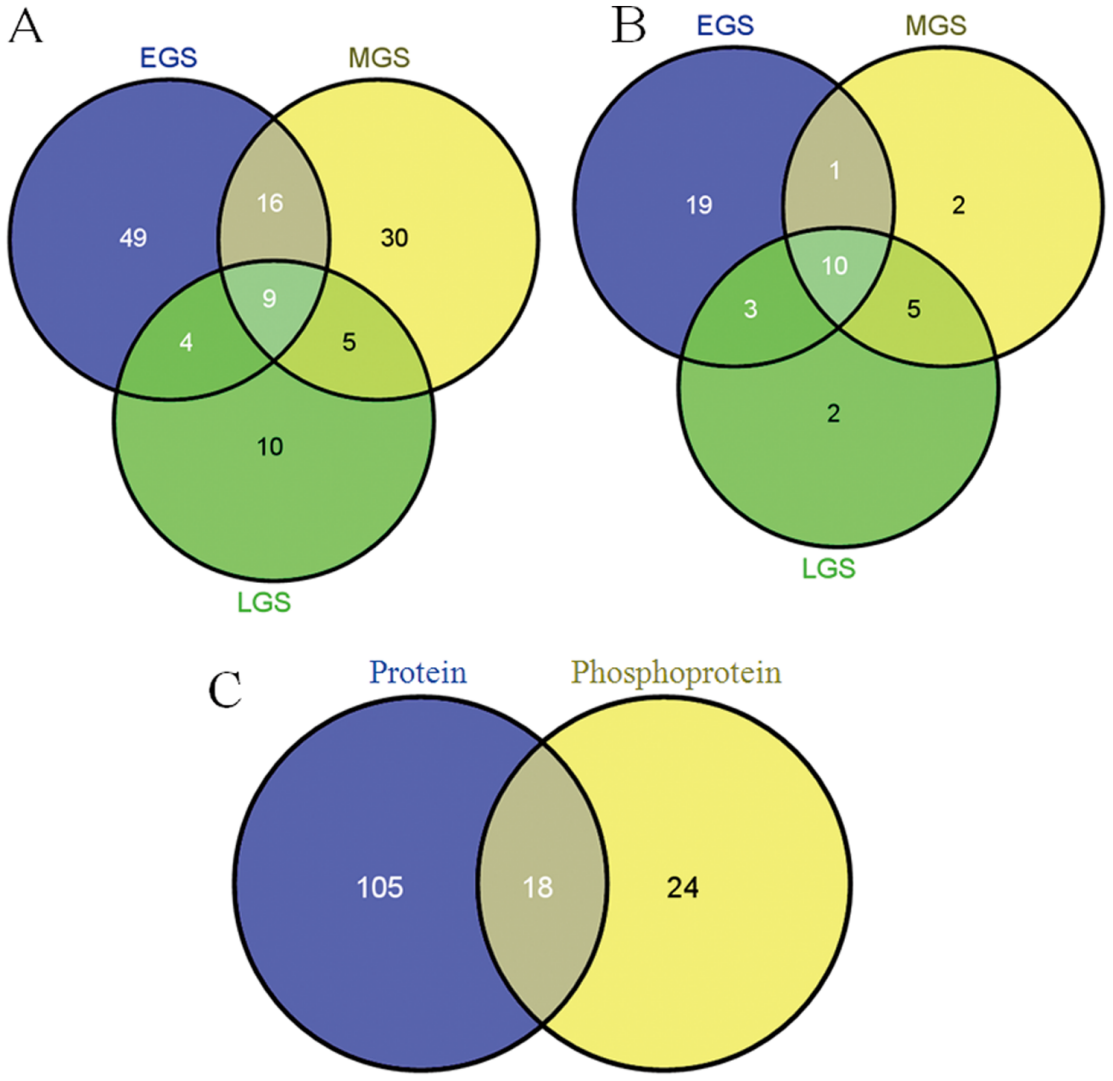
Differentially expressed protein and phosphoprotein for superior and inferior spikelets. A: Code numbers of proteins at three stages. B: Code numbers of phosphoproteins at three stages. C: Comparative proteins and phosphopoteins.

Some of the protein and phosphoprotein spots were further analyzed by MALDI-TOF-MS or LC-ESI-MS/MS. At the end, most of them were successful identified (Table 1 and 2). In all, 81, 61, and 29 unique proteins and 33, 18, and 20 phosphoproteins in EGS, MGS and LGS, respectively, were identified. Among them, 9 proteins and 10 phosphoproteins showed significant differences between SS and IS for all grain-filling stages (Fig. 6A–B). Eighteen proteins, such as 14-3-3, ADPase and Pyruvate orthophosphate dikinase (PPDK), showed significant differences in expression abundance, as well as phosphorylation level, between SS and IS (Fig. 6C), indicating their possible important roles in regulating the grain-filling.

**Table 1:** List of protein spots with differential expressions between superior and inferior spikelets in 3 grain-filling stages.

PN^a^	SN^b^	An^c^	Protein name	Score	Mr(KDa)/pI d	MP.^e^	CT^f^										
							EGS			MGS						LGS	
**1- Metabolism**																	
**1.1- Sugars conversion**																	
**1**	63^g^	B9FUS5	Similar to Xylose isomerase	243	53.8/5.42	3	DR				–						–
**2**	271^h^	Q8H3Q7	Xylose isomerase	37	53.5/5.66	2	–				UR						–
**3**	234^h^	Q75K72	Putative beta-1,3-glucanase’	37	34.7/6.37	5	UR				–						–
**4**	213^h^	Q10DZ9	Phosphoglucomutase, cytoplasmic 2	274	62.9/5.63	7	–				DR						–
	66^g^	Q10DZ9	Phosphoglucomutase, cytoplasmic 2	243	54.7/4.93	2	–				DR						–
**5**	273 h	Q9AUQ4	Phosphoglucomutase	239	62.9/5.63	17	–				DR						–
**6**	268^h^	Q33AE4	Phosphoglucomutase	53	66.1/6.68	2	–				DR						–
**7**	78^g^	Q93X08	Similar to UTP–glucose-1-phosphateuridylyltransferase	108	51.8/5.43	2	–				DR						–
	276^h^	Q93X08	UDP-glucose pyrophosphorylase	355	51.7/5.59	15	–				DR						–
	293^h^	Q93X08	UDP-glucose pyrophosphorylase	143	51.7/5.59	10	–										DR
**8**	69^g^	Q0J8G4	Similar to Fructokinase	430	35.9/5.02	4	–				DR						–
**9**	13^g^	Q8H8T0	UDP-L-arabinose mutase 1	324	40.1/8.21	3	–				DR						–
**1.2-Starch synthesis**																	
**10**	14^g^	B8XED2	ADP-glucose pyrophosphorylase large subunit	556	58.2/5.55	4	DR		–			DR					
	50^g^	B8XED2	ADP-glucose pyrophosphorylase large subunit	355	58.2/5.55	3	UR		DR			–					
	49^g^	B8XED2	ADP-glucose pyrophosphorylase large subunit	189	58.2/5.55	2	DR		–			–					
**11**	97^g^	B8XEC0	ADP-glucose pyrophosphorylase large subunit	410	58.2/5.71	3	DR		DR			–					
**12**	141^g^	P15280	Similar to Glucose-1-phosphateadenylyltransferase small subunit	420	53.2/5.87	3	–		DR			DR					
**13**	215^h^	Q69T99	Glucose-1-phosphate adenylyltransferase	500	54.8/6.67	2	–		DR			–					
	232^h^	Q69T99	Glucose-1-phosphate adenylyltransferase	52	54.8/6.67	5	DR		–			–					
**14**	98^g^	A8QXE7	Granule-bound starch synthase	347	63.4/8.77	3	DR		–			–					
**15**	226^h^	Q7X834	Pullulanase	954	106.4/6.30	4	DR		–			–					
	220^h^	Q7X834	Pullulanase	208	106.4/6.30	4	DR		–			–					
**1.3-Pyruvate,orthophosphate dikinase (PPDK)**																	
**16**	83^g^	Q6AVA8	Pyruvate,orthophosphate dikinase	377	103.6/5.98	3	DR	–					DR				
	84^g^	Q6AVA8	Pyruvate,orthophosphate dikinase	222	103.6/5.98	3	DR	–					DR				
	85^g^	Q6AVA8	Pyruvate,orthophosphate dikinase	367	103.6/5.98	3	DR	DR					–				
**1.4- Amino acid**																	
**17**	188^g^	Q9XEA8	Cysteine synthase	547	34.4/5.35	5	DR	DR							–		
**18**	252^h^	Q0DZE3	Phenylalanine ammonia-lyase	231	75.5/6.49	21	DR	–							–		
	253^h^	Q0DZE3	Phenylalanine ammonia-lyase	158	75.5/6.49	16	DR	–							–		
**19**	222^h^	Q93WM3	Asparaginyl-tRNA synthetase, cytoplasmic 1	559	62.5/5.90	2	UR	–							–		
**20**	77^g^	O49218	Mthylmalonate semi-aldehyde dehydrogenase	271	57.5/5.99	3	DR	UR							UR		
**21**	121^g^	Q8VXC4	Glycine rich RNA binding protein	297	19.6/6.59	2	–	UR							–		
**22**	104^g^	Q84V24	Aspartate aminotransferase	282	46.0/5.90	4	–	DR							–		
**23**	284^h^	Q6ZHC3	Putative aspartate-tRNA ligase	100	60.8/6.32	6	–	DR							–		
**24**	46^g^	Q7XXQ8	Putative RNA-binding protein	144	41.8/5.08	2	–	DR							–		
**25**	74^g^	Q65XK0	Similar to Ketol-acid reductoisomerase	288	62.7/6.01	3	–	DR							DR		
**26**	282^h^	Q2QLY5	Methionine synthase	208	84.5/6.30	13	–	DR									
**27**	12^g^	Q5JNB0	Similar to Cysteine synthase	176	42.1/6.28	2	–	–							DR		
**28**	140^g^	Q651F0	Similar to 3-dehydroquinate synthase-like protein	73	47.5/8.05	2	–	–							DR		
**1.5- Nucleotides**																	
**29**	111^g^	P49027	Guanine nucleotide-binding protein beta subunit-like protein	394	36.7/5.97	3	UR	DR							–		
**30**	112^g^	Q2R1V8	NAD-dependent epimerase/dehydratasefamily protein	284	42.5/5.75	3	DR	–							–		
**31**	265^h^	Q5Z6P9	Putative RAD23 protein	23	43.0/4.77	2	–	UR							–		
**32**	82^g^	Q84P58	Adenosine kinase-like protein	201	40.6/5.57	3	–	–							UR		
**1.6- Lipid**																	
**33**	119^g^	Q69YA2	Similar to 3-oxoacyl-[acyl-carrier-protein]synthase I	99	49.5/6.76	2	UR	–							–		
**1.7- Glycolysis**																	
**34**	235^h^	Q6H6C7	Phosphoglycerate kinase	611	42.1/5.86	19	DR	–							–		
	236^h^	Q6H6C7	Phosphoglycerate kinase	117	42.1/5.86	7	UR	–							–		
	277^h^	Q6H6C7	Phosphoglycerate kinase	448	42.1/5.86	13	–	DR							–		
	292^h^	Q6H6C7	Phosphoglycerate kinase	519	42.1/5.86	22	–	–							DR		
**35**	231^h^	Q75K90	Phosphoglycerate kinase	184	32.4/9.92	9	DR	–							–		
**36**	79^g^	Q53P96	Fructose-bisphosphate aldolase class-I	101	39.6/6.85	2	DR	UR							–		
	269^h^	Q53P96	Fructose-bisphosphate aldolase class-I	51	39.3/7.30	6	–	DR							–		
**1.8- Tricarboxylic acid (TCA) pathway**																	
**37**	144^g^	Q9ASP4	Dihydrolipoamide dehydrogenase family protein	331	53.0/5.71	3	UR	–							–		
**38**	80^g^	Q7XDC8	Cytoplasmic malate dehydrogenase	393	35.9/5.75	3	DR	–							–		
		Q7XDC8	Cytoplasmic malate dehydrogenase	393	35.9/5.75	3	–	DR							–		
**39**	151^g^	Q7F280	Similar to NADP-isocitrate dehydrogenase	209	46.4/6.34	2	–	DR							–		
**40**	275^h^	Q6H7M1	Putative fumarylacetoacetate hydrolase	79	47.1/5.94	5	–	DR							–		
**41**	274^h^	Q6Z702	Putative 3-isopropylmalate dehydratase large subunit	104	55.5/7.33	12	–	DR							–		
**1.9- Pentose phosphate pathway (PPP)**																	
**42**	219^h^	Q0J8A4	Glyceraldehyde-3-phosphate dehydrogenase	337	36.7/6.81	4	–	UR							–		
**43**	65^g^	Q84ZY2	Putative transketolase	319	80.6/6.12	3	–	–							DR		
**1.10- Fermentation**																	
**44**	175^g^	Q10MW3	Similar to Pyruvate decarboxylase isozyme 3	497	65.8/5.53	5	DR	DR							–		
**45**	18^g^	Q6ZBH2	Alcohol dehydrogenase superfamily, zinc-containing protein	483	40.0/6.03	3	–	–							DR		
**1.11- Nitrogen**																	
**46**	286^h^	Q338N8	Alanine aminotransferase	170	52.6/6.65	11	–	DR							–		
**47**	157^g^	P14655	Glutamine synthetase,chloroplastic	265	49.8/6.18	2	UR	DR							–		
**48**	2^g^	P14656	Glutamine synthetase cytosolic isozyme	340	39.4/5.51	3	–	DR							DR		
**2-Protein synthesis and destination**																	
**2.1- Protein folding and modification**																	
**49**	105^g^	Q9LWT6	Similar to 60 kDa chaperonin	289	64.3/5.60	2	DR	–							–		
	47^g^	Q9LWT6	Similar to 60 kDa chaperonin	253	64.3/5.60	2	DR	–							–		
**50**	6^g^	Q53LQ0	Protein disulfide isomerase	226	57.0/4.95	2	DR	–							–		
	5^g^	Q53LQ0	Protein disulfide isomerase	259	57.0/4.95	2	DR	–							–		
**51**	7 g	Q84P99	Heat shock-related protein	337	45.0/5.02	2	DR	–							DR		
**52**	9^g^	Q0DJB9	Similar to Stromal 70 kDa heat shock-related protein	479	48.7/4.57	3	DR	–							DR		
**53**	129^g^	Q943K7	Heat shock protein Hsp70 family protein	440	71.3/5.10	4	DR	DR							–		
**54**	103 g	Q53P60	Similar to Heat shock 70 kDa protein	446	73.1/5.49	4	DR	UR							DR		
**55**	240^h^	Q2QU06	60 kDa chaperonin alpha subunit	21	61.1/5.21	2	UR	–							–		
**56**	224^h^	Q5Z907	T-complex protein 1, epsilon subunit	535	59.1/6.00	4	DR	–							–		
	257^h^	Q5Z907	Putative T complex protein	35	59.1/6.02	2	DR	–							–		
**57**	88^g^	Q69Y99	Putative chaperonin 21 precursor	389	26.4/7.71	2	UR	–							–		
**58**	223^h^	C7J006	Cupin region domain containing protein	406	45.3/6.50	8	UR	–							–		
**59**	218 h	C7IZM0	Proteinase inhibitor I4, serpin family protein	264	29.2/5.47	2	–	DR							–		
**60**	261^h^	Q5QLP0	Putative heat shock protein 82	34	70.7/5.15	2	–	DR							–		
**61**	214^h^	Q9AUV8	Phosphorylase	951	106.2/5.94	11	–	DR							–		
	279^h^	Q9AUV8	Phosphorylase	602	106.2/5.94	24	–	DR							–		
	280^h^	Q9AUV8	Phosphorylase	434	106.2/5.94	18	–	DR							–		
**62**	270^h^	Q6Z7L1	Putative dnaK-type molecular chaperone	104	72.9/5.59	8	–	DR							–		
**63**	262^h^	Q0D9G9	Heat shock protein HtpG 10	52	92.8/4.98	5	–	DR							–		
**64**	216^h^	Q9AYD4	HSP-70 cofactor 5	332	36.5/4.72	5	–	DR							–		
**65**	291^h^	Q2QV45	70 kDa heat shock protein	79	74.0/5.25	7	–	–							DR		
**2.2- Protein synthesis**																	
**66**	247^h^	Q8H3I3	Putative 40S ribosomal protein	52	33.0/5.08	5	UR	–							–		
**67**	94^g^	P29545	Elongation factor 1 beta’	316	23.8/4.86	3	DR	DR							–		
**68**	71^g^	Q40680	Similar to Elongation factor 1 beta 2	269	24.9/4.36	2	–	DR							–		
**69**	73^g^	O24182	Endosperm lumenal binding protein	264	73.7/5.30	4	–	–							DR		
	128^g^	O24182	Endosperm lumenal binding protein	368	73.7/5.30	3	–	–							DR		
	130^g^	O24182	Endosperm lumenal binding protein	264	73.7/5.30	3	DR	–							–		
**2.3-Proteolysis**																	
**70**	32^g^	Q6H852	Proteasome subunit alpha type 2	268	25.8/5.39	3	UR	–							–		
**71**	143^g^	Q5SNJ4	Similar to Mitochondrial processing peptidase	249	54.1/6.69	2	UR	–							–		
**2.4-Protein storage**																	
**72**	227^h^	Q0JJ36	Glutelin	488	56.2/8.87	5	DR	–							–		
**73**	228^h^	Q10JA8	Glutelin	385	56.0/8.53	7	DR	–							–		
**74**	233^h^	Q0DH05	Alpha-globulin	66	21.0/7.50	4	DR	–							–		
**3-Signal transduction**																	
**75**	70^g^	Q7XTE8	Similar to 14-3-3-like protein GF14-6	246	30.0/4.76	3	UR	UR							UR		
**76**	38^g^	Q6YZA9	Germin-like protein 3	354	19.6/8.89	2	DR	–							–		
**4-Disease/defense**																	
**77**	28^g^	Q7XUY5	Bet v I allergen family protein	238	17.3/4.75	2	UR	–							–		
**78**	191^g^	Q7XEL9	Similar to Chitinase 2	328	31.2/4.48	4	UR	–							–		
	266^h^	Q7XEL9	Similar to Chitinase 2	71	31.2/4.68	5	–	–							DR		
**79**	23^g^	Q7XCK6	Chitinase	273	28.0/6.28	2	UR	–							–		
**80**	30^g^	Q5WMX0	Similar to ChitinaseIII	219	32.8/6.08	2	UR	DR							UR		
**81**	42^g^	Q0DRV6	Superoxide dismutase	143	15.3/5.71	2	UR	–							–		
**82**	242^h^	Q0DJ64	Superoxide dismutase	164	25.0/7.02	9	UR	–							–		
**83**	243^h^	Q0J7H9	Glyoxalase	39	32.5/5.67	5	UR	–							–		
**84**	92^g^	Q9FR35	Peroxiredoxin-2C	186	17.3/5.58	2	UR	–							–		
**85**	241^h^	Q65XA0	Dehydroascorbate reductase	281	23.6/6.21	10	UR	–							–		
**86**	256^h^	Q5QNE8	Putative 4-methyl-5(B-hydroxyethyl)-thiazol monophosphate biosynthesis enzyme	32	45.1/6.30	2	UR	–							–		
**87**	197^h^	Q5Z9Y8	Late embryogenesis abundant protein	58	27.9/4.40	4	DR	DR							–		
**88**	115^g^	Q0JL44	Sgt1	431	41.0/4.97	4	UR	–							–		
**89**	27^g^	Q8H8U5	Glutathione S-transferase	322	34.0/8.86	3	UR	–							–		
**90**	36^g^	Q9FE01	L-ascorbate peroxidase	276	27.2/5.21	3	DR	–							–		
	91^g^	Q9FE01	L-ascorbate peroxidase	434	27.2/5.21	3	–	UR							UR		
**91**	145^g^	Q9SXP2	Nad-dependent formate dehydrogenase	345	41.4/6.87	3	–	DR							–		
**92**	248^h^	Q6H660	Putative stress-induced protein sti1	180	64.9/6.38	16	DR	–							–		
	283^h^	Q6H660	Putative stress-induced protein sti1	227	64.9/6.38	15	–	DR							–		
**93**	136^g^	Q9AVA6	Putative selenium binding protein	306	51.3/5.73	3	–	DR							–		
**94**	288^h^	Q6ZK46	Putative early embryogenesis protein	48	58.0/8.44	2	–	UR							–		
**5-Transporters**																	
**95**	62^g^	Q0JKB4	Similar to ATP synthase beta chain, mitochondrial precursor	280	59.6/6.10	2	DR		–						DR		
	3^g^	Q0JKB4	Similar to ATP synthase beta chain, mitochondrial precursor	595	59.6/6.10	3	UR		DR						–		
**96**	258^h^	Q6ZG90	Putative ATP synthase	31	27.3/6.93	6	DR		–						–		
**97**	225^h^	Q93W07	Vacuolar ATPase B subunit	488	54.0/5.20	2	DR		–						–		
**98**	229^h^	Q6Z8K7	Putative H(+)-transporting ATP synthase	20	26.2/5.06	3	UR		–						–		
**99**	198^h^	Q651T8	Putative vacuolar proton-ATPase	234	68.4/5.30	15	DR		–						–		
**100**	156^g^	Q01859	Mitochondrial F1-ATPase beta subunit	22	59.1/6.30	2	DR		UR						–		
**101**	190^g^	P0C521	ATP synthase F0 subunit 1	471	55.5/5.85	3	–		DR						–		
**102**	303 h	Q5N7P8	ATP synthase subunit beta	135	45.2/5.43	2	–		–						DR		
**6-Cell growth/division**																	
**103**	1^g^	P0C539	Actin	408	42.2/5.72	3	DR		–						–		
**104**	177^g^	Q8S3Q3	Similar to Possible apospory-associated like	97	38.1/5.10	3	DR		–						–		
**105**	122^g^	P35681	Similar to Translationally controlled tumor protein	140	19.0/4.51	2	DR		–						–		
**106**	263^h^	Q10CU1	Tubulin beta-7 chain	25	49.8/4.87	4	DR		DR								
**7-Secondary metabolism**																	
**107**	166^g^	Q9XGP7	Similar to Caffeoyl-CoA O-methyltransferase 2	514	27.9/5.11	3	DR		–						–		
**108**	87^g^	Q9XJ19	Similar to Caffeoyl-CoA 3-O-methyltransferase	396	29.0/5.21	3	–		–						UR		
**109**	127^g^	Q75LL5	Putative strictosidine synthase	17	54.2/8.75	2	UR		–						–		
**110**	95^g^	Q9SXV0	Similar to Cytochrome C oxidase subunit 6b	52	19.1/4.27	2	UR		–						–		
**111**	61^g^	Q10Q21	Similar to Cytochrome C reductase-processing peptidase subunit I, MPP subunit I, P55	160	58.9/5.49	2	DR		–						–		
	51^g^	Q10Q21	Similar to Cytochrome C reductase-processing peptidase subunit I, MPP subunit I, P55	387	58.9/5.49	3	DR		DR								
**112**	25^g^	Q84T92	Chalcone isomerase	153	24.0/5.15	2	–		–						UR		
**9 -Photosynthesis**																	
**113**	26^g^	Q6Z3V7	Putative Photosystem I reaction center subunit IV	178	15.5/9.64	2	DR		–					–			
**114**	8^g^	P93431	Ribulose-1,5-bisphosphate carboxylase/oxygenase activase	498	48.1/5.85	3	UR		UR					–			
	86^g^	P93431	Ribulose-1,5-bisphosphate carboxylase/oxygenase activase	352	48.1/5.85	3	–		DR					–			
**115**	48^g^	Q7X9A7	Putative rubisco subunit binding-protein alpha subunit precursor	717	61.5/5.36	5	DR		–					–			
**116**	68^g^	Q2QW49	Ribulose bisphosphate carboxylase large chain precursor	300	56.5/9.04	3	–		DR					–			
	108^g^	Q2QW49	Ribulose bisphosphate carboxylase large chain precursor	389	56.5/9.04	4	–		–					DR			
**117**	133^g^	Q40701	23 kDa polypeptide of photosystem II	336	27.1/9.54	3	–		DR					–			
**118**	285^h^	Q8S6F2	Putative rbcL; RuBisCO large subunit from chromosome 10 chloroplast insertion	361	52.9/6.92	16	–		DR					–			
**119**	304^h^	Q5QLG3	Phosphoribulokinase/uridine kinase-like	89	46.1/6.90	3	–		–					UR			
**10- Unclassified**																	
**120**	244^h^	Q8H684	OSEYA1	52	34.1/5.11	4	DR		–					–			
**121**	230 h	Q0DUA3	Os03g0197300 protein	28	68.2/5.71	3	DR		–					–			
**122**	180^g^	Q53RR0	Mov34/MPN/PAD-1 family, putative	16	139.3/9.93	2	UR		–					–			
**123**	123^g^	B8BKE8	Hypothetical protein OsI_36050	23	17.1/11.15	2	DR		–					–			

Note:

a proteins number,

b protein spots number correspond to those on 2-DE gels shown in Fig. 3;

c accession number;

d theoretical MW (kDa) and pI.

e match peptides;

f changes on protein spots in superior spikelets compared to inferior spikelets; UR: protein upregulated in inferior spikelets as compared to superior spikelets; DR: protein downregulated in inferior spikelets as compared to superior spikelets; EGS: early grain-filling stage, MGS: mid-grain-filling stage, LGS: late grain-filling stage;

g protein spot identified by MALDI-TOF-MS;

h protein spot identified by LC-ESI-MS/MS.

**Table 2:** List of phosphoprotein spots with differential expressions between superior and inferior spikelets in 3 grain-filling stages.

PN^a^	SN^b^	An^c^	Protein name	Score	Mr(KDa)/pI e	MP.^f^	CT^g^		
							EGS	MGS	LGS
**1 Metabolism**									
**1.1 Sugars conversion**									
**1**	P 1	Q33AE4	Phosphoglucomutase	106	66.1/6.70	9	–	DR	DR
**2**	P5	Q9AUQ4	Phosphoglucomutase	276	62.9/5.60	22	–	–	UR
**3**	P 9	Q93X08	UDP-glucose pyrophosphorylase	134	51.7/5.60	2	–	DR	–
**4**	P26	Q6ZBH2	Putative sorbitol dehydrogenase	263	39.3/6.47	18	DR	–	–
**5**	P63	Q8S9Z2	Putative dTDP-glucose 4,6-dehydratase	39	44.3/6.25	5	DR	–	–
**6**	P61	Q9AUV8	Phosphorylase	352	106.2/5.94	17	UR	–	DR
	P62	Q9AUV8	Phosphorylase	258	106.2/5.94	18	UR	DR	–
**1.2 Starch synthesis**									
**7**	P6	Q5VNT5	Glucose-1-phosphate adenylyltransferase	353	57.0/5.70	15	DR	–	–
	P68	Q5VNT5	Glucose-1-phosphate adenylyltransferase	278	57.0/5.70	19	–	DR	DR
**8**	P80	A8QXE7	Granule-bound starch synthase	347	63.4/8.77	3	DR	DR	–
**1.3 Pyruvate, phosphate dikinase (PPDK)**									
**9**	P77	Q6AVA8	Pyruvate, phosphate dikinase	794	93.6/5.50	26	DR	–	DR
	P79	Q6AVA8	Pyruvate, phosphate dikinase	794	93.6/5.50	26	DR	–	DR
**1.4 Amino acid metabolism**									
**10**	P19	Q5JNB0	Cysteine synthase	33	41.8/6.70	3	DR	–	–
**11**	P28	Q0INQ6	Serine hydroxymethyltransferase	52	50.7/7.69	5	DR	–	–
**12**	P30	Q338N8	Alanine aminotransferase	147	52.6/6.70	10	DR	–	UR
	P51	Q338N8	Alanine aminotransferase	291	52.6/6.65	21	DR	DR	UR
**13**	P37	Q0DPW7	3-isopropylmalate dehydrogenase	168	41.2/5.40	15	DR	–	–
**14**	P22	Q6H7M1	Putative fumarylacetoacetate hydrolase	79	47.1/5.94	8	DR	–	–
**1.5 Nucleotides**									
**15**	P35	Q6K1R5	Putative adenosine kinase	154	37.0/5.16	14	–	DR	UR
**1.6 Lipid**									
**16**	p52	Q6Z0I4	Putative enoyl-ACP reductase	60	39.1/8.68	7	DR	–	DR
	P70	Q6Z0I4	Putative enoyl-ACP reductase	123	39.1/8.70	10	–	UR	UR
**1.7 Glycolysis**									
**17**	P4	Q5QMK7	Phosphoglycerate mutase	423	60.8/5.68	20	DR	–	–
	P60	Q5QMK7	Phosphoglycerate mutase	310	60.8/5.68	19	DR	DR	UR
**18**	P25	Q6H6C7	Phosphoglycerate kinase	28	42.1/5.86	3	DR	–	–
**19**	P72	A6MZY0	Phosphoglycerate kinase	29	18.0/4.70	2	–	DR	UR
**20**	P67	Q10P35	Enolase 2, putative, expressed	436	47.9/5.50	14	–	DR	–
	P74	Q10P35	Enolase 2, putative,	26	47.9/5.50	2	–	–	UR
**2 Protein synthesis and destination**									
**2.1 Protein synthesis**									
**21**	P58	Q6ZI53	Elongation factor Tu	156	50.4/6.64	10	DR	–	–
**22**	P56	Q6Z058	Putative Luminal binding protein 5	154	73.5/5.22	6	DR	–	–
**23**	P31	Q5W6H1	Putative DNA-binding protein GBP16	154	43.2/7.08	12	DR	–	UR
**2.2 Protein folding and modification**									
**24**	p55	Q0J0U8	Heat shock protein	148	80.1/5.06	17	UR	–	–
**25**	p54	Q5QLP0	Putative heat shock protein 82	118	70.7/5.15	13	UR	–	–
**26**	P3	Q8H903	60 kDa chaperonin	343	60.8/5.87	23	–	–	UR
**27**	P53	Q2QV45	70 kDa heat shock protein	682	74.0/5.25	34	UR	DR	UR
**28**	P57	Q6Z7B0	Dnak-type molecular chaperone Bip	522	73.3/5.19	22	DR	UR	–
	P69	Q6Z7B0	Dnak-type molecular chaperone	427	73.3/5.20	13	–	DR	UR
**2.3 Storage protein**									
**29**	P32	Q0JJ36	Glutelin	488	56.2/8.87	5	UR	–	–
	p50	Q0JJ36	Glutelin	320	56.2/8.87	7	UR	–	–
**30**	P33	Q10JA8	Glutelin	385	56.0/8.53	7	UR	–	–
	P36	Q10JA8	Glutelin	30	56.0/8.53	2	DR	–	–
**31**	P49	Q6YTX6	Seed protein	15	27.4/7.64	4	DR	–	–
**3 Signal transduction**									
**32**	P16	Q10E23	14-3-3 protein	279	29.2/4.90	8	DR	DR	DR
**33**	p17	Q7XTE8	Similar to 14-3-3-like protein GF14-6	246	30.0/4.76	3	DR	DR	DR
**4 Transporters**									
**34**	P29	Q8S7T5	ATP synthase subunit alpha	220	55.1/6.25	14	DR	–	–
**35**	P59	Q651T8	Vacuolar proton-ATPase	175	68.4/5.34	15	DR	–	–
**36**	P14	Q6H7I9	ATP-dependent Clp protease proteolytic subunit	27	31.9/7.20	2	DR	–	–
**37**	P66	Q8S6F3	ATP synthase subunit beta	430	54.0/5.50	20	–	DR	
**5 Cell growth/division**									
**38**	P27	Q0PVB0	Alpha-tubulin	71	49.6/5.06	5	DR	–	–
**39**	P11	Q6ZK46	Early embryogenesis protein	171	58.0/8.40	5	–	DR	UR
**6 Secondary metabolism**									
**40**	P12	Q19BJ6	Flavone O-methyltransferase	125	39.7/5.66	11	DR	–	–
	P64	Q19BJ6	Flavone O-methyltransferase	41	39.7/5.70	5	–	DR	DR
**7 Photosynthesis**									
**41**	P20	Q6Z8F4	Phosphoribulokinase	110	44.8/6.02	11	DR	–	–
**8 Unknown**									
**42**	P48	Q0JDG9	Os04g0404400 protein	15	31.2/5.02	2	DR	–	UR

Note:

a phosphoproteins number,

b phosphoprotein spots number correspond to those on 2-DE gels shown in Fig. 4;

c score;

d theoretical MW (kDa) and pI.

e Match peptides;

f changes of phosphoprotein spots in inferior spikelets as compared to superior spikelets, UR: phosphoprotein upregulated in inferior spikelets as compared to superior spikelets; DR: phosphoprotein downregulated in inferior spikelets as compared to superior spikelets; EGS: early grain-filling stage, MGS: mid-grain-filling stage, LGS: late grain-filling stage.

### Function Classification of Differentially Expressed Proteins and Phosphoproteins for SS and IS

**Figure 7:**
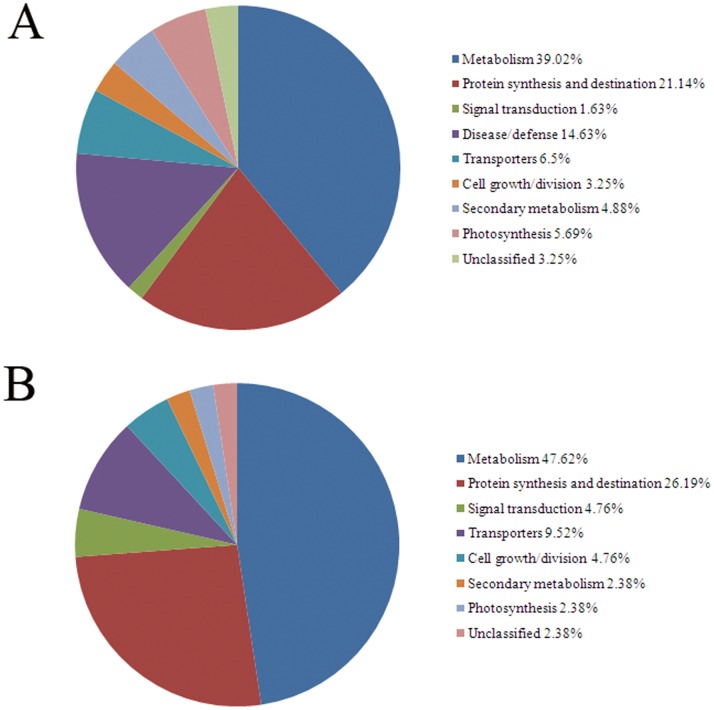
Function classifications of identified proteins (A) and phosphoproteins (B).

According to genetic ontology, the identified proteins and phosphoproteins were classified into 8 functional categories, i.e., metabolism, protein synthesis/destination, signal transduction, disease/defense response, transporter, cell growth/division, secondary metabolism and photosynthesis (Fig. 7, Table 1 and 2). Those proteins and phosphoproteins without appropriate genetic ontology terms and/or could not be classified into the 9 categories were marked as “unknown” in this report.

Of the 123 identified proteins, 39.02% were associated with the metabolism, and 21.14% the protein synthesis/destination (Fig. 7A). For the 43 phosphoproteins, 46.62% were grouped with the metabolism and 26.19% the protein synthesis/destination (Fig. 7B). The metabolism and protein synthesis/destination functions seemed crucial in differentiating the grain-fillings between SS and IS.

**Figure 8:**
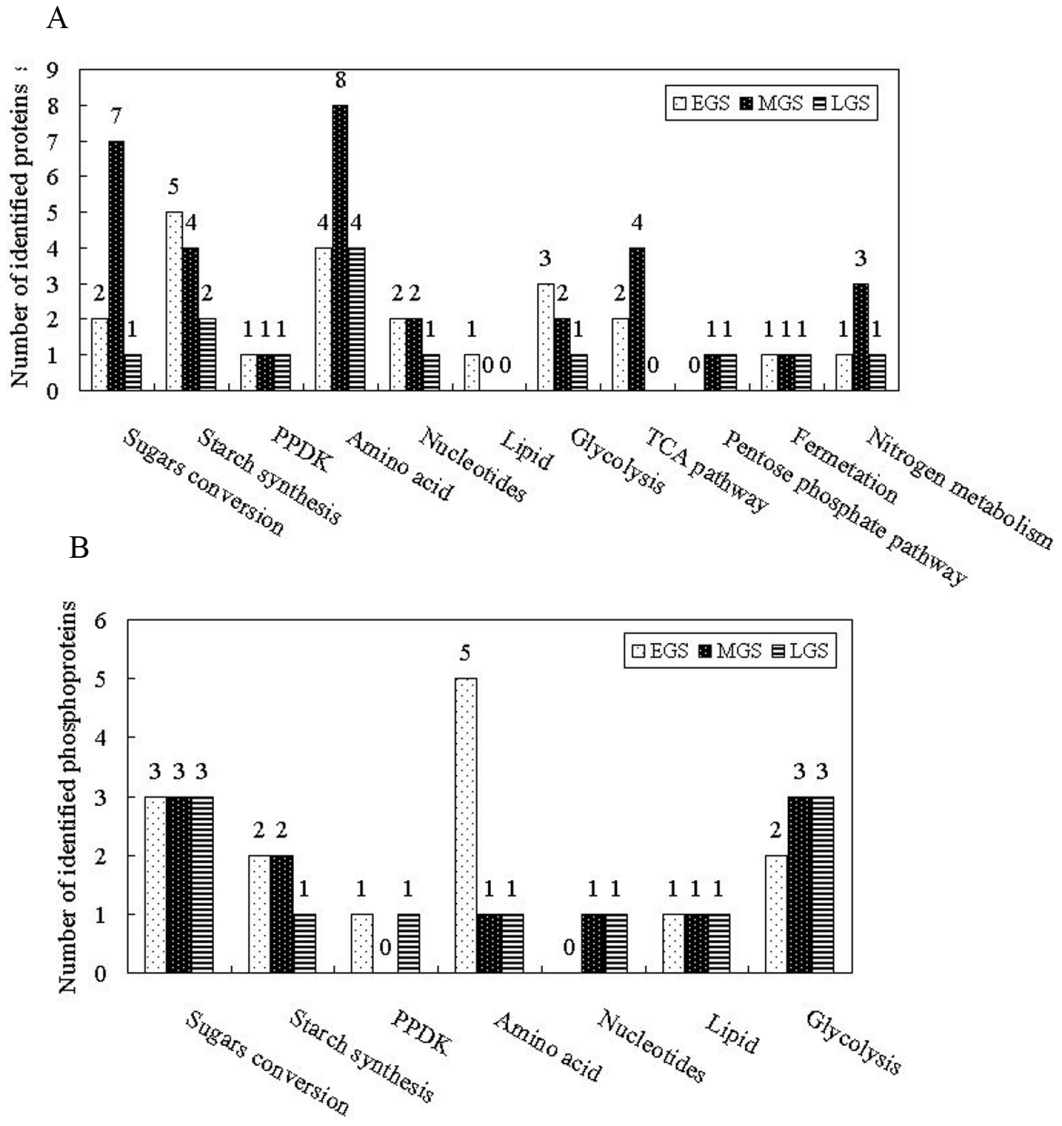
Function classifications of identified metabolism-related proteins (A) and phosphoproteins (B).

To analyze the metabolic processes of SS and IS at different grain-filling stages, the proteins involved were further divided into 11 subcategories including sugar conversion, starch synthesis, PPDK, amino acid metabolism, nucleotide metabolism, lipid metabolism, glycolysis, tricarboxylic acid (TCA) pathway, pentose phosphate pathway (PPP), fermentation and nitrogen metabolism (Fig. 8A, Table 1). And, the phosphoproteins were divided into 7 subcategories that included sugar conversion, starch synthesis, PPDK, amino acid metabolism, nucleotide metabolism, lipid metabolism and glycolysis (Fig. 8B, Table 2). Plant PPDK was initially discovered in C4 leaves, and recently found to be abundant in developing wheat [19], maize [20] and rice endosperm [21] but the function of PPDK in seed development remains to be elucidated. Therefore, PPDK proteins were organized as an independent category.

### Western Blotting of Differentially Expressed Proteins

**Figure 9:**
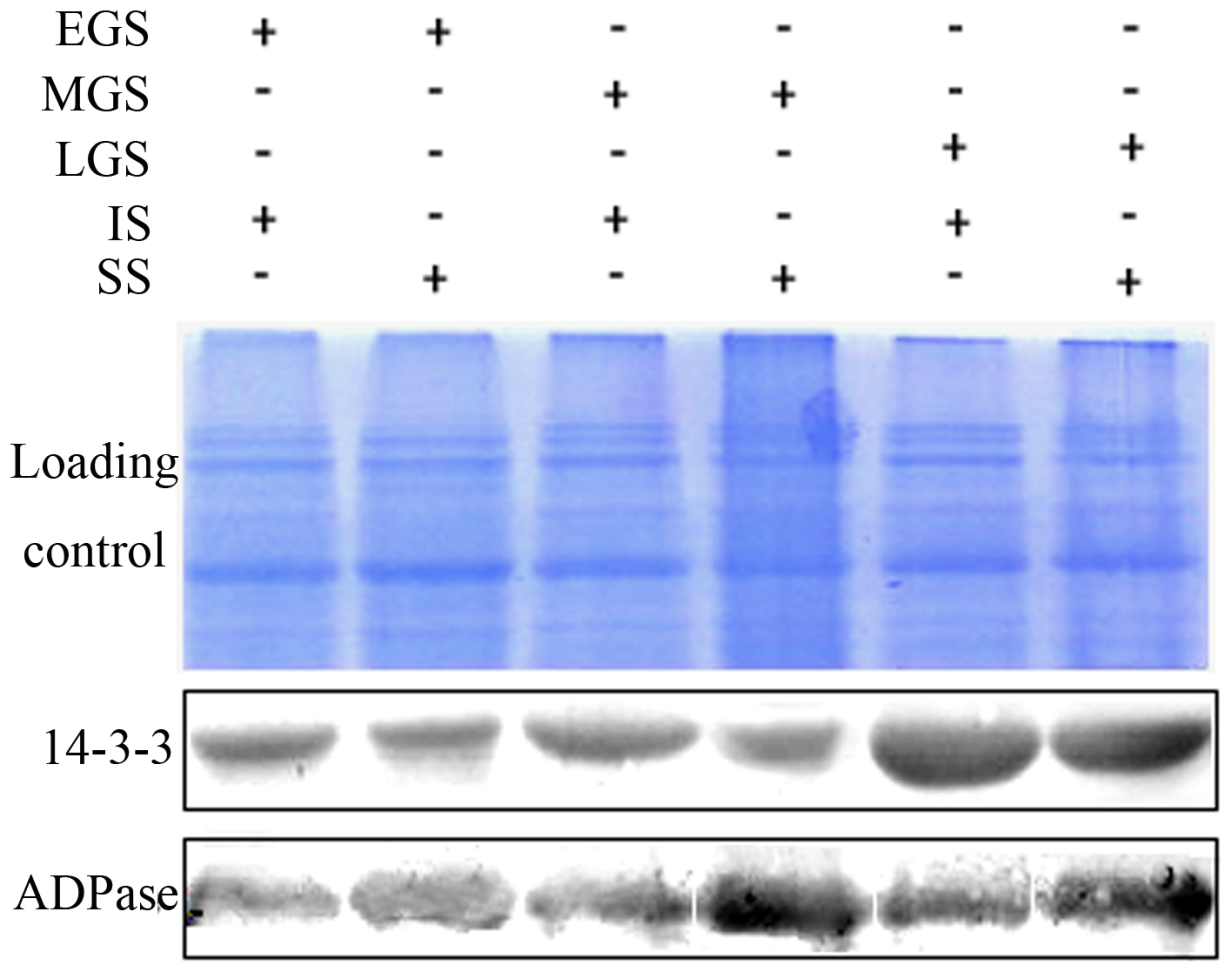
Western blotting for comparing 14-3-3 protein and ADPase expressions of superior spikelets (SS) and inferior spikelets (IS) at three grain-filling stages. Gels were divided into two parts at a molecular mass of approximately 66-3-3 protein and ADPase. EGS, MGS and LGS represent early, mid-, and late grain-filling stage, respectively, of endosperm development stages of SS and IS.

Western blotting was conducted to confirm the differentially expressed proteins detected by 2-DE gels using the mouse antisera as the primary and the HRP-antimouse IgG as the secondary antibodies against 14-3-3 and ADPase (Fig. 9). The 14-3-3 spots showed unregulated and ADPase spots downregulated on IS as compared to SS for all three grain-filling stages.

## Discussion

### Physiological Differences between SS and IS

Both grain size and weight of SS were greater than those of IS. It is commonly believed that the poor IS grain-filling was due to a limited carbohydrate supply [22], [23]. However, the present study showed that the soluble carbohydrates in grains on IS was higher than SS during EGS and MGS, indicating that the carbohydrate supply should be more abundant in IS than SS. Then, in LGS, the carbohydrate content became lower on IS than SS. It might be caused by a nutritional scarcity and/or plant senescence. Apparently, the difference of SS and IS grain-filling must be a highly complex process, and to clarify it would require a further exploration on its molecular mechanism. Thus, from the standpoint of protein expression and phosphorylation, we took the proteomic approach to investigate the differences in them between SS and IS during the grain-filling stages of the rice.

### Different Proteomic Characteristics of SS and IS Displayed at Three Grain-filling Stages

A comprehensive proteomic analysis with 2-DE was performed to show a total of 156 protein spots with differential abundances by more than 1.5-fold between SS and IS at three grain-filling stages. All of them (represented by 123 unique proteins) were successfully identified by MALDI- TOF- MS or LC-ESI-MS/MS. To verify that some of the differentially expressed proteins were indeed regulated by a post-translational mechanism, such as protein phosphorylation, the 2-DE gels of SS and IS at those grain-filling stages were stained with Pro-Q Diamond dye. The proteomics coupled with the Pro-Q Diamond staining allowed a positive identification of phosphorylated proteins as well as the changes occurred, as Pro-Q Diamond binds specifically to the phosphate moiety of phosphoproteins with high sensitivity and linearity [24], [25]. As a result, 54 phosphoprotein spots (represented by 43 unique phosphoproteins) with significant differences, as assessed by their fluorescence intensities, were identified to be induced by the phosphorylation (≥1.5-fold).

Among the identified proteins and phosphoproteins, 18 proteins showed significant differences in both expression abundance and phosphorylation. Of the 18 proteins, 11 differed defferently at a same grain-filling stage. For example, the expression abundance of 14-3-3 in IS was higher than that in SS at all stages. Contrarily, the phosphorylation was lower in IS. Therefore, the protein expression and the phosphorylation were indeed two different and independent properties in the grain-filling process. Base on the proteomic analysis, the number of differentially expressed proteins or phosphoproteins in EGS was larger than that in MGS or LGS. It indicated that the difference between SS and IS occurred in EGS was most crucial for the grain-filling. In all three grain-filling stages, merely 16 proteins (13%) and 10 phosphoproteins (23.3%) showed significant differences between SS and IS. It suggested that the molecular mechanisms varied at different grain-filling stages as well.

The differentially expressed proteins and phosphoproteins were further annotated by a GO analysis. According to the molecular functions and biochemistry, they were likely to be involved in the biological metabolic processes such as metabolism, protein synthesis and destination, cell growth/division, disease/defense, transporters and signal transduction. A MicroRNA analysis between SS and IS identified 189 differentially expressed microRNAs involved in similar biological metabolic processes [26]. And, our study also found the largest number of the identified proteins or phosphoproteins to be associated with metabolism. This suggested that metabolism could be the most critical function that differentiated the grain-fillings between SS and IS.

### Low Activity of Starch Synthesis-related Enzymes and Poor IS Grain-filling

**Figure 10:**
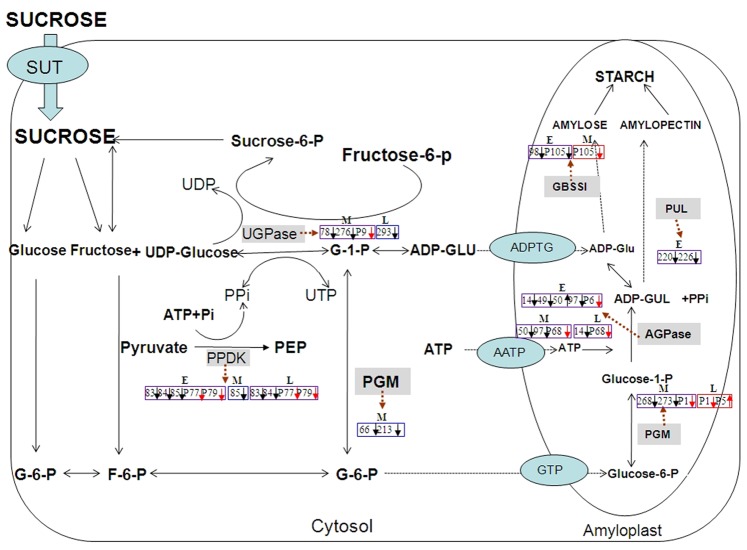
Difference on starch biosynthesis pathways between superior spikelets (SS) and inferior spikelets (IS). Model of starch biosynthetic pathway according to Reference [28]. Blue pane represents differentially expressed proteins, red pane differed phosphorylation, and purple pane either differentially expressed proteins or differed phosphorylation. E, M and L represent early, mid-, and late grain-filling stage, respectively, of endosperm development stages of SS and IS. Black arrow represents proteins downregulated or upregulated in IS as compared to SS. Red arrow represents phosphoproteins downregulated or upregulated in IS as compared to SS. PGM: Phosphoglucomutase; UGPase: UDP-glucose pyrophosphorylase; AGPase: ADPglucose pyrophosphorylase; ADPGT: ADPglucose transporter; AATP: plastidic ATP transporter; GTP: Glucose-6-Phosphate transporter; GBSS: Granule bound starch synthase; Pul: Pullulanase; PPDK: Pyruvate, orthophosphate dikinas.

As previous reports indicated, low activities of the starch synthesis enzymes, such as SuSase, AGPase, StSase, and SBE, closely related to the poor grain-filling of IS [10]–[12], [27]. Recently, the DNA microarray analysis showed that there were more enhanced expression profiles and higher transcript levels on a group of genes related to the starch metabolism on SS than IS [17]. Our comparative proteomic study indicated that most of such proteins were downregulated on IS at different grain-filling stages. It seemed to confirm, at protein expression level, that the low activity of the starch synthesizing enzymes was the main reason that caused the poor grain-filling of IS. On the basis of our proteomic data and other published results in literature [28], we proposed a model for the defference regulatory networks of starch synthesis between SS and IS filling (Fig. 10). A difference in the enzymatic activity and carbohydrate content between SS and IS was indeed evident in our study which led to the following conclusion. During EGS, the lower abundance of starch synthesis proteins (i.e., AGPase, GBSS and Pul) on IS, i.e., AGPase, granule-bound starch synthase (GBSS) and pullulanase (Pul), slowed the starch accumulation, as compared to SS. And, in MGS, the downregulated expression of sugar converting proteins, i.e., phosphoglucomutase (PGM) and UDP-glucose pyrophosphorylase (UDPase), and starch synthesizing proteins (AGPase) further slowed the grain-filling. Finally, in LGS, IS had a low protein abundance of UDPase and AGPase accompanied by a low carbohydrate content leading to the low starch accumulation in IS.

The starch synthesis enzyme activity has been shown to be regulated by the protein phosphorylation [29], [30]. In the present study, 5 starch synthesis proteins, i.e., PGM, UDPase, phosphorylase, AGPase and GBSS, were stained with Pro-Q Diamond dye to reveal the differences between SS and IS on phosphorylation. These proteins have been identified as phosphoproteins previously. PGM activity can be significantly increased by the signaling kinase p21-activated kinase 1 (PAK1)-mediated phosphorylation of PGM selectively on threonine 466 [31]. A resent study found that the serine phosphorylation of Wx protein played an important role in regulating GBSS activity at the posttranslational level [32]. Consequently, phosphorylation could induce changes in the starch synthesis enzyme activity. Moreover, protein phosphorylation is considered a prerequisite for the formation of the starch synthesizing protein complex (SSPC)in cereal endosperm [29], [33], [34]. And, in wheat and maize amyloplast, the starch synthesis enzymes, such as AGPase, SBE, and StSase, can assemble into a SSPC to function in the starch biosynthesis [20], [35]. The formation of SSPC in cereal endosperm explains how the mutations, which affect specific starch biosynthesis enzymes, have pleiotropic effects on other enzymes in the starch synthesis pathway. Different levels of phosphorylation in starch synthesis enzymes might lead to varied expressions of SSPC in SS and IS as well.

There are also other SSPC enzymes, such as PPDK found in maize and the wheat amyloplast SSPC [20] that lack defined roles in starch synthesis. For example, we identified 3 distinctive PPDK isoforms in SS and IS. It catalyzes the reversible conversion of pyruvate, ATP and Pi into phosphoenolpyruvate (PEP), AMP, and PPi. A recent proteomic study showed the differentially expressed PPDK isoforms in developing wheat [19], maize [20] and rice endosperm [21], indicating its close relationship with the seed development. A protein expression analysis on maize endosperms showed that PPDK might play an important role in the starch-protein balance through the inorganic pyrophosphate-dependent restriction of ADP-glucose synthesis [19]. Hence, it was speculated that the different abundance of protein expressions and phosphorylation’s of PPDK might contribute to the varied activities of the starch synthesis enzymes in SS and IS. The hypothesis has yet been proven, and PPDK’s role in the grain-filling remains to be further studied.

### Slowed Glycolysis, TCA Cycle and Alcohol Fermentation vs. Reduced Energy Supply and Building Materials for IS

The central carbon metabolism (glycolysis and TCA cycle) provides energy, cofactor regeneration, and building materials for the interconversions and synthesis of metabolites [36], [37]. The change in the concentration of the metabolites is thus used as an indicator for the biological process. Likewise, the dynamic changes of the protein expression during the grain development of rice reflected the progress of the glycolysis and TCA cycle, which peaked in EGS or MGS. The rice grains were filled from EGS to MGS going through the sink establishment in EGS by endosperm cell enlargement, and thereafter, the starch synthesis. Completion of the prominent glycolysis and TCA cycle in EGS and MGS require an adequate supply of ATP and the synthesis of cellular components essential for the cell enlargement and starch synthesis [21]. In our study, the expression abundance of most of the identified proteins relating to the glycolysis and TCA cycle was found to be lower in the grains on IS than SS. The key glycolysis enzymes were downregulated during EGS and MGS, and those associated with the TCA cycle in MGS. The results suggested that the slowed glycolysis and TCA cycle in IS during EGS and MGS reduced the progress of the endosperm cell enlargement and starch synthesis, which were detrimental to the sink establishment in the grains.

Three glycolysis proteins, phosphoglycerate mutase (PGAM), phosphoglycerate kinase (PGK) and enolase, were detected by means of Pro-Q Diamond staining. PGK was also identified in oilseed rape by the same staining method, and its phosphopeptide by LC-MS/MS/MS [25]. PGAM phosphorylation was detected in arabidopsis seeds [38]. And, the enolase was phosphorylated on a single tyrosine [39]. These enzymes encountered varied phosphorylation on SS and IS, indicating that the glycolysis in IS could be artificially altered through phosphorylation manipulation. Our earlier phosphoproteomic work [15] showed that the addition of ABA could indeed change the phosphorylation of PGK and enolase in IS to promote the grain-filling.

During LGS, the two key enzymes in alcohol fermentation, ADH and transketolase, were downregulated in IS. The fermentation is a two-step process that includes the branching from the glycolysis pathway at pyruvate with concomitant oxidization of NADH to NAD+, and then, the generating of ATP anaerobically [40]. In a typical development of seeds or bulky organs (such as wheat seeds and potato tubers), the internal oxygen concentration is greatly reduced [41]–[43]. The upregulation of alcohol fermentation pathway is very important for maintaining an appropriate ATP level for the starch synthesis under low oxygen tension [21]. Thus, downregulation of the alcohol fermentation in IS at LGS might also cause a reduction on ATP limiting the normal synthesis of starches.

Consequently, it appeared that the changes occurred on the expression and phosphorylation of the proteins associated with glycolysis, TCA cycle and/or alcohol fermentation could bring about the poorer-than-expected grain-filling on IS.

### Difference on N Metabolisms between SS and IS

Adequate supply of N is crucial for rice grain-filling, as it directly affects the carbon assimilation and biomass accumulation in the grains [44], [45]. In the present study, alanine aminotransferase (AlaAT) and glutamine synthetase (GS) associated with N metabolism were identified with 3 protein spots. A previous report showed *AlaAT* gene expressed at high levels in the developing rice grains, and suggested its involvement in N metabolism during the maturation of the rice grains [46]. GS activity in rice grains was found to be positively related to the grain yield [47]. The expressions of GS and AlaAT were both downregulated on IS in MGS and LGS, possibly resulted from an insufficient N supply in the paddy soil that led to an accelerated aging of the rice plants at the later stages. Our earlier proteomic studies showed that an appropriate N fertilization at the late growth stage could significantly promote the GS protein accumulation on IS [45], as suggested by the abovementioned hypothesis. Thus, the low expressions of AlaAT and GS during MGS and LGS could be additional factors affecting the grain-filling of IS.

### Low Expression Abundance of Cell Growth/Division Proteins and Grain-filling of IS

Abundant evidences have shown that the low endosperm cell division rate and cell count can cause poor grain-filling on IS [2], [4], [13]. Our study identified 4 proteins and 2 phosphoproteins relating to the IS cell growth/division. The translationally controlled tumor protein (TCTP) was identified and downregulated on IS in EGS. TCTP has been implicated in important cellular processes, such as cell growth, cell cycle progression and malignant transformation, as well as the protection of cells against various stress conditions and apoptosis [3]. TCTP has also been shown to interact with elongation factors, eEF1A [48] and tubulin [49]. The eEF1A functions in the coordinated regulation of the multiple cellular processes including growth, division, and transformation [50]. Tubulin has previously been associated with the cell division during embryogenesis and in a number of environmental and developmental signals [51], [52]. Recently, arabidopsis TCTP (*AtTCTP*) was found to function in the ABA-mediated stomatal movement, in addition to regulation of the plant growth [49]. In general, TCTP is considered to play a role in (1) cell cycle control, (2) growth factor, (3) ABA response, and (4) interaction with elongation factors, eEF1A and tubulin. Interestingly, two putative TCTP interaction proteins, elongation factor 1 beta and tubulin, were also downregulated and had a very similar downregulation profile like TCTP in EGS on IS. It suggested a possible involvement of this pathway in the IS cell division as well. In addition, tubulin can be stained by Pro-Q Diamond dye to differentiate phosphorylation between SS and IS. And, the phosphorylation sites of tubulin were determined during the development of oilseed rape seeds, and its phosphorylation was considered to be important for the cell division [25].

GLP have been found in various organs (i.e., leaves, roots, and floral tissues) in plants, and under different physiological conditions (e.g., seed germination, stress, and pathogen attack) [47], [53]. In plum fruits, two GLPs genes were identified, and their transcripts were upregulated with the increase of endogenous auxin, indicating that GLPs could be part of the auxin signal network that mediates the cell division and expansion [54]. Auxin is an important signal in the cereal endosperm development [55], and, low auxin leads to a low cell division on rice IS [13]. In the present study, the GLP expression was lower on IS in EGS, indicating a possible difference existed in the auxin signal pathways between SS and IS that led to a lowered cell division of IS.

Previous reports showed that the application of ABA and auxin (IAA) in EGS significantly increased the endosperm cell division rate and cell count on IS [4]. Collectively, it was concluded that, in addition to regulating the cell count of IS, TCTP with eEF1A and tubulin functioned in the ABA-mediated cell development, and that GLP in the auxin signal pathway.

### 14-3-3 Protein and Grain-filling of IS

In our study, 14-3-3 proteins from SS and IS differed significantly in regard to both protein expression abundance and phosphorylation. The proteins are a family of conserved regulatory molecules expressed in all eukaryotic cells [56]–[58]. They are capable of binding a multitude of functionally diverse signaling proteins, including kinases, phosphatases, and transmembrane receptors [59]. A recent proteomic study on soybean and oilseeds also found the proteins highly expressed during the seed development with an involvement in the signaling and metabolic pathways [25], [60]. Furthermore, 14-3-3 are phosphopeptide-binding proteins with its phosphorylation detected in oilseed rape [25]. However, no *in vivo* phosphorylation of the proteins in rice grains has been identified thus far.

Stained with Pro-Q Diamond dye, 14-3-3 protein showed phosphorylation levels different between SS and IS. Based on the following analysis, it appeared that 14-3-3 protein played a critically important role in the poor IS grain-filling. Firstly, in a proteomic interaction analysis on wheat and barley endosperms, the largest category of the 14-3-3 binding proteins was associated with the carbohydrate metabolism that included plastidic enzymes for starch synthesis and modification [61]. And, some of the binding proteins, such as GBSS, PPDK and ADP glucose pyrophosphorylase large subunit, were also detected in our study showing a lower expression abundance on IS than SS. Secondly, the previous studies reported an inhibitive ability of 14-3-3 protein on the activity of starch synthesis enzymes [61]–[63], while Sehnke et al. found the reduction of the 14-3-3 proteins correlated to the starch granule formation with an increase in the starch content on their study of arabidopsis leaf starch [62]. Previously, we also observed the appropriate addition of ABA to be favorable for starch synthesis on IS. In the proteomic study that followed, a downregulation of the 14-3-3 protein under the ABA treatment was clearly demonstrated [15]. Therefore, it seemed apparent that a highly expressed 14-3-3 protein was detrimental to the starch formation and accumulation. And, the protein expression of 14-3-3 on IS was shown by the 2-DE and western blotting results to be higher than SS in all three grain-filling stages. Lastly, 14-3-3 protein participates in various signal transduction and regulatory processes by interacting with diverse target proteins in a sequence-specific and phosphorylation-dependent manner [64], [65]. Since the activity regulation of a certain enzymes can be a two-step process of phosphorylation and complex formation with 14-3-3 protein [56], [66], our finding of the differentiated 14-3-3 phosphorylation between SS and IS suggested another potential factor in the complex mechanism.

As the data shown, it seemed likely the differently expressed abundance and phosphorylation of 14-3-3 to be the root cause for the low activity of starch synthesis enzymes. And in turn, the resulting poor grain-filling of IS was observed. Nonetheless, the underlying mechanism of 14-3-3 protein in regulating the starch synthesis still remains to be fully uncovered.

This study identified 123 proteins proteomically different between SS and IS of the rice. The proteins’ possible roles in regulating the grain-filling were categorized based on the biological protein-interaction or reverse genetic functions of the genes. The classification illustrated the vast complexity of the grain-filling process, which might include metabolism, proteins synthesis and destination, signal transduction, transporters and cell growth/division.

For the first time, the Pro-Q Diamond Fluorescence Staining Method was applied to enable positive differentiation of the 43 phosphoproteins between SS and IS. Nonetheless, due to availability of methodologies, the current study could not identify all the phosphoproteins in the grains. Methods with a highly efficient phosphoprotein enrichment capability, such as TiO_2_ and Ti^4+^-MAC, could be of help in obtaining an improved profiling for the grain-filling of rice.

## Materials and Methods

### Plant Material and Sampling

The experiments were carried out at the Experimentation Station of Fujian Agriculture and Forestry University, Fuzhou, Fujian, China (119.280E, 26.080 N) during the rice growing season. The large-panicle rice cultivars, Jinhui No. 809 (*Oryza sativa* Indica), was used. The seeds were immersed in a water bath at room temperature for 24 h, and subsequently germinated under moist conditions at 37°C for 48 h. The germinated seeds grew in a paddy field. The seedlings at the 5-leaf stage were transplanted with a spacing of 0.15×0.15 m and one seedling per hill. Each 4×4 m planting plot received a fertilization of 225 kg N ha^−1^, 112.5 kg P_2_O_5_ ha^−1^, and 180 kg K_2_O ha^−1^. The phosphorus fertilizer was used as the basal, and the potassium for the top dressing. The field soil was sandy loam with available N, phosphorus, and potassium of 200.1, 147.3 and 208.2 mg kg^−1^, respectively.

Five hundred panicles that headed on a same day were tagged. The flowering date and position of each spikelet on the tagged panicles were recorded. The duration of anthesis from the first spikelets to the last on a panicle was 7 d. SS and IS were distinguished according to the previous report [2]. Thirty tagged panicles were sampled every 5 days from 0 to 35 day after flowering (DAF, the day was accounted from the first day of flowering). Spikelet samples for protein extraction were obtained 5, 10, 15, 20, 25, and 30 DAF. They were frozen in liquid N immediately after collection, and stored at −80°C.

### Determinations of Grain Weight, Soluble Carbohydrate and Sucrose Content

Ten tagged panicles were sampled every 5 d from heading to maturity. SS and IS samples were separated, blanched in an oven at 105°C for 15 min, and dried at 80°C to a constant weight. Thirty grains were used for soluble carbohydrate and sucrose determinations. The samples were dried at 70°C to a constant weight, and dehulled for further analysis. Soluble carbohydrate and starch contents of SS and IS were determined as described by Yoshida et al [67].

### Protein Extraction, 2-DE and Statistical Analyses

Proteins in the developing SS were extracted after harvested at 5, 10 and 15 DAF, while at 20, 25 and 30 DAF for IS, when their endosperm development were in EGS, MGS and LGS, respectively. Total protein was extracted in the same manner as reported previously [15]. Briefly, 1.0 g of spikelets was mixed with 0.5 g polyvinyl pyrrolidone, ground to fine powder in a mortar, and subsequently immersed in liquid N. The sample was suspended in a pre-cooled, 10% trichloroacetic acid in acetone containing 0.07% β-mercaptoethanol solution, and kept at −20°C overnight. The thawed sample was then centrifuged at 15,000 rpm for 30 min at 0–4°C. After decanting the supernatant, the precipitate was washed with a pre-cooled, 100% acetone containing 0.07% β-mercaptoethanol solution followed by centrifugation at 15,000 rpm for 30 min at 0–4°C. These procedures were repeated approximately every 8 h until the supernatant was achromatic. Then, the precipitate was dried under vacuum to yield sample pellet. The dried pellet was dissolved in a lysis buffer containing 8 mol/L urea, 4% CHAPS, 40 mmol/L Tris and 65 mmol/L DTT. The mix was homogenized by using ultrasound for 20–25 min, followed by centrifugation at 15,000 rpm for 15 min at 0–4°C. The supernatant was collected and stored at −80°C for proteomic analysis. The protein content was determined by using a previously described method [68].

Isoelectric focusing was carried out with 1.3 mg protein per strip using 24 cm long Immobiline Dry-strips pH 4–7 Linear (GE Biosciences). The protein were loaded onto an IPG strip and focused using an Ettan IPGphor 3 System (GE Healthcare) with a ceramic manifold as follows: gradient to 500 V for 1 h; gradient to 1000 V for 2 h; gradient to 8 KV for 3 h; step and hold at 8 KV for 3 h. The strips were then equilibrated upon gentle agitation on a stirrer twice for 15 min in an equilibration buffer (0.1 M Tris-HCl pH 8.8, 6 M urea, 30% (v/v) glycerol, and 2% (w/v) SDS) containing, firstly, 65 mM DTT, and, then, 2.5% (w/v) iodoacetamide. SDS-PAGE was performed in 26×20 cm, 10.5% (v/v) polyacrylamide gels using an Ettan Dalt 6 Multiple Apparatus (GE Biosciences) at 15°C. Fifteen mA per gel was used during running time until the dye front reached the end of gel. Gels were then stained with colloidal Coomassie Blue G-250 using at least 300 ml of staining solution per gel [69]. The gels were also stained with Pro-Q Diamond phosphoprotein gel stain (Molecular probes, Invitrogen) by fixing in 50% ethanol/10% acetic acid overnight, washing three times (15 min each) in deionized water, incubating in Pro-Q Diamond stain for 120 min in the dark, destaining with three washes of 20% acetonitrile in 50 mM sodium acetate (pH 4.0) and washing twice in deionized water. Images were acquired with Typhoon Variable Model Images Trio+ (GE Healthcare) using 532 nm excitation and a 580 nm band pass emission filter. After fluorescent images were acquired, the gels were stained with Coomassie Blue G-250 again. Gels were then scanned with an ImageScanner (GE Healthcare), and analyzed with Imagemaster 5.0 (GE Healthcare). Protein spots displaying ≥1.5-fold increase or decrease in abundance on average (p<0.05) were selected for protein identification.

### In-gel Protein Digestion

In-gel protein digestion was performed with modifications as described previously [70]. In brief, the protein spots from same 2D-Gels were finely excised and transferred to siliconized 0.5 mL Eppendorf tubes. Each gel piece was rinsed twice with deionized water, destained in 25 mM ammonium bicarbonate in water/acetonitrile (50/50) solution (a 1∶1 solution of 30 mM potassium ferricyanide and 100 mM sodium thiosulfate), and then equilibrated in 50 mM ammonium bicarbonate to pH 8. After dehydrated with acetonitrile in a Speed-Vac Centrifuge (Thermo Fisher Scientific, Waltham, MA), the gel spots were rehydrated in a minimal volume of trypsin (Promega, USA) solution (12.5 µg/mL in 25 mM NH_4_HCO_3_) and incubated at 37°C overnight. The supernatants were transferred to a 200 µl microcentrifuge tube, and the gels were extracted once with the buffer (67% acetonitrile containing 2.5% trifluoroacetic acid).

### Mass Spectrometric Analysis

#### MALDI-TOF-MS analysis

The peptide extract was combined with the supernatant of the gel spots and dried thoroughly in a Speed-Vac. The extract from the protein digestion (tryptic peptides) was re-suspended in 5 µl of 0.1% trifluoroacetic acid. In 1∶1 ratio, the peptide samples were mixed with a saturated solution of α-cyano-4-hydroxy-trans-cinnamic acid in 30% acetonitrile containing 0.1% trifluoroacetic acid. One µl of the aliquots were, then, spotted onto the stainless steel sample target plates. Protein spots of interest were cut from the gels and sequenced by Tandem mass spectrometry (MS/MS) with a fuzzy logic feedback control, a Reflex III MALDI-TOF System (Bruker) equipped with a delayed ion extraction. Both MS and MS/MS analyses were carried out with air as the collision gas using 1-kV collision energy.

#### LC-ESI-MS/MS analysis

For the protein samples that were not successfully identified by MALDI-TOF- MS, the extracted tryptic fragments were further characterized by using a LTQ-XL Mass Spectrometer (Thermo Scientific, USA) coupled with an Accera System (Thermo Fisher Scientific). A biobasic C18 column (100×0.18 mm; particle size: 5 um, Thermo Scientific, USA) was used to separate the digested proteins. Solvent A was 0.1% HCO_2_H mixed in water, and Solvent B was 0.1% HCO_2_H mixed in CH_3_CN. The gradient was held at 2% Solvent B for 2 min, and increased linearly up to 90% Solvent B over the course of 60 min. The peptides were eluted from a C18 column at a flow rate of 160 µl/min and then electrosprayed directly into an LTQ mass spectrometer using a spray voltage of 3.5 kV and at the capillary temperature of 275°C. Acquisitions were performed in data-dependent MS/MS scanning mode.

### Database Searching

MS data were analyzed, and peak lists generated using Flexanalysis version 2.0 (Bruker). MS peaks were selected between 800 and 3,000, and filtered with a signal to noise ratio greater than 15 to exclude masses derived from trypsin autolysis. Search parameters allowed for one missed tryptic cleavage site, the carbamidomethylation of cysteine, possible oxidation of methionine, and a precursor ion mass tolerance of 100 ppm. Peptide masses were searched using Protein Prospector Mascot (http://www.matrixscience.com). *O. sativa* database was used for protein identification. The probability score at 95% confidence level as calculated using the software was the criterion for correct identification.

The raw data obtained from LC-ESI-MS/MS were used to search on the *O. sativa* database from Uniprot (http://www.uniprot.org/) using Proteome Discover 1.2 (Thermo Fisher Scientific). The carbamidomethylation of cysteine was set as a fixed modification, while the oxidation of methionine, a variable modification. Trypsin was the proteolytic enzyme and one missed cleavage was allowed. The search parameters included a precursor ion mass tolerance of 2.5 Da, a fragment mass tolerance of 0.5 Da, and a maximum missed cleavage of 2.

### Western Blotting Analysis

Western blotting on the 1-DE gels was performed as described previously. Rabbit antisera to 14-3-3 and ADPase were obtained from Huada Proteomic Research Center, Beijing, China. In brief, the 1-DE gels were transferred to NC membranes for 3 h at 50 V in a transfer buffer (48 mM Tris, 39 mM glycine, 1.3 mM SDS and 20% methanol) at 4°C. The membranes were blocked with 5% BSA, and then, incubated with rabbit antisera to 14-3-3 or ADPase as the primary antibodies. Horseradish peroxidase (HRP) conjugated goat anti-rabbit antibodies were used as the secondary antibodies. Antibody-tagged protein spots were detected by 3, 3′-diaminobenzidine (DAB).
